# Pear phytobezoar as a rare cause of small bowel obstruction: a case report

**DOI:** 10.3389/fmed.2025.1620356

**Published:** 2025-09-11

**Authors:** Kuanyong Yu, Wenjun Feng, Liyang Liu, Chuanyang Cao, Guanghui Qiang

**Affiliations:** Department of General Surgery, Nanjing Jiangbei Hospital, Nanjing, China

**Keywords:** small intestinal obstruction, phytobezoar, pear, enterotomy, laparoscopy

## Abstract

A phytobezoar is an accumulation of indigestible fruit and vegetable fibers in the gastrointestinal (GI) tract. Small bowel obstruction (SBO) caused by phytobezoars is infrequent, and pear-induced instances are extremely rare. We present a 45-year-old woman with a 5-day history of colicky abdomen discomfort, nausea, vomiting, abdominal distention, and no bowel movements. She had two cesarean sections but no prior GI issues and had eaten an extensive quantity of pears 2 days before symptom onset. A physical examination showed discomfort, distension, and decreased bowel sounds in the abdomen. Laboratory testing was normal, but computed tomography (CT) revealed dilated small intestine loops and a transition point in the right lower abdomen. Conservative treatment, which included fasting, intravenous fluids, antibiotics, and nasogastric decompression, failed. A 30 mm × 30 mm pear bezoar blocking the terminal ileum was removed through a 4-cm enterotomy during laparoscopy. The enterotomy was closed transversely, and the patient healed normally, remaining symptom-free 3 months later. This case emphasizes the value of staying aware of phytobezoar-induced SBO in individuals’ patients who have not previously undergone stomach surgery. Early dietary history, imaging characteristics, and timely surgical intervention are crucial. Individualized dietary counseling can aid in preventing recurrence in at-risk patients.

## Introduction

Small-bowel obstruction (SBO) is a common surgical emergency with high morbidity and fatality rates (1). It happens when the normal flow of intestinal contents is mechanically obstructed, which can be caused by extrinsic or intrinsic intestinal wall lesions, as well as intraluminal blockages (2). Adhesions, hernias, tumors, and inflammatory bowel disease are the most common causes, accounting for nearly 90% of cases (3).

Bezoars masses of indigestible food or fiber are a rare cause of SBO, contributing for 0.4%–4.0% of cases (4). Phytobezoars, which are created from poorly digested fruit and vegetable fibers, are the most prevalent variety, while pear-induced bezoars are especially rare. While bezoar-induced SBO is frequently associated with previous gastric surgery, it can also occur in patients without a surgical history (5).

Excessive consumption of fibrous foods, poor chewing skills, gastrointestinal (GI) motility issues, and other lifestyle variables all contribute to the condition. Clinical suspicion, a complete food history, and imaging findings are required for a prompt diagnosis. Depending on the degree of the obstruction and the response to initial treatment, management may consist of conservative medicinal therapy or medical surgery is performed.

Here, we report a case of pear-induced SBO in a 45-year-old with no prior GI surgery, emphasizing the significance of early detection, appropriate care, and preventive dietary counseling.

## Case report

**FIGURE 1:**
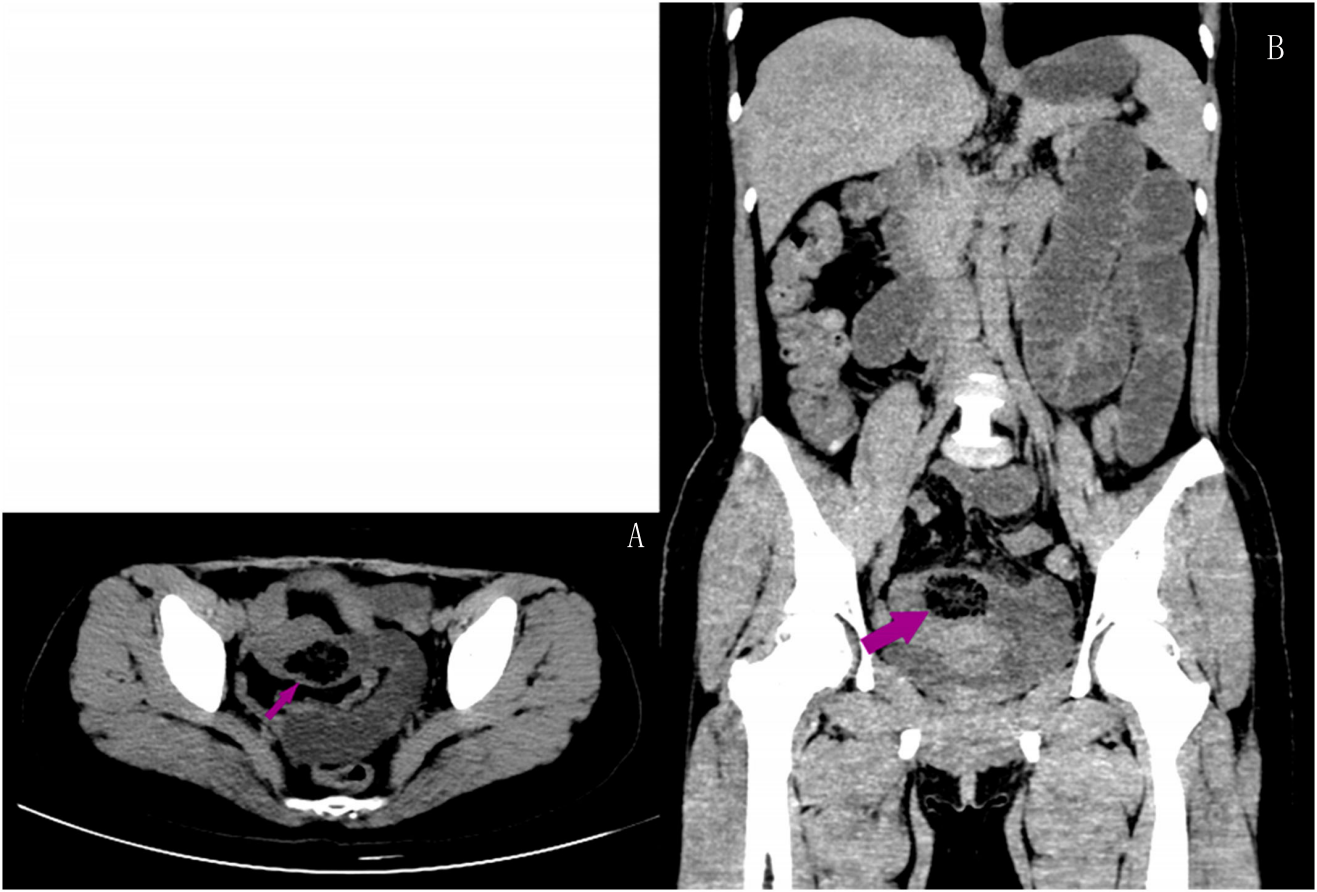
Axial **(A)** and coronal **(B)** abdominal CT scan revealing an oval mottled-appearing mass suggesting a bezoar (arrow), with proximal small bowel distension.

**FIGURE 2:**
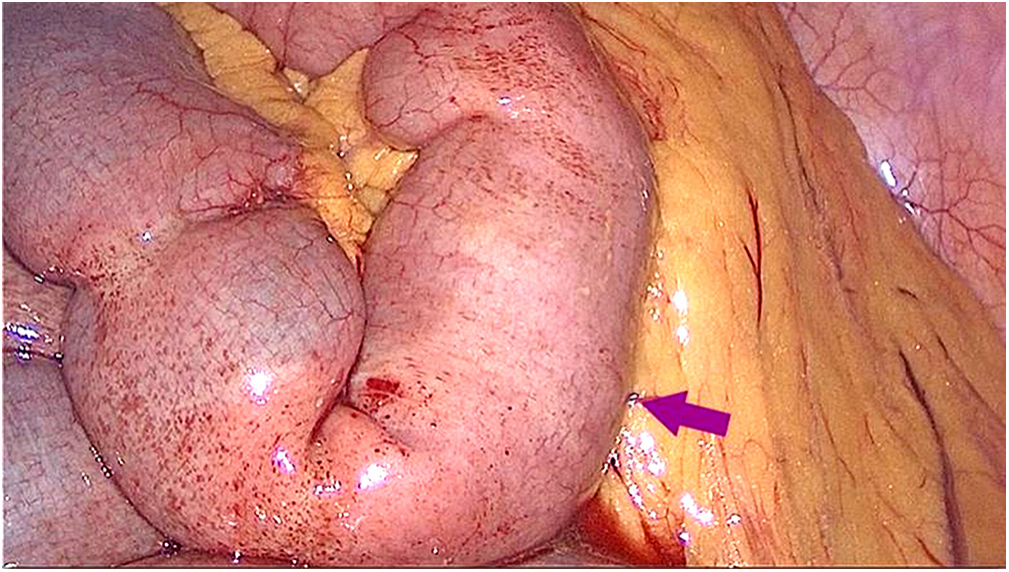
Laparoscopic examination revealed a columnar foreign body impacted within the small intestinal lumen approximately 60 cm from the ileocecal valve.

**FIGURE 3:**
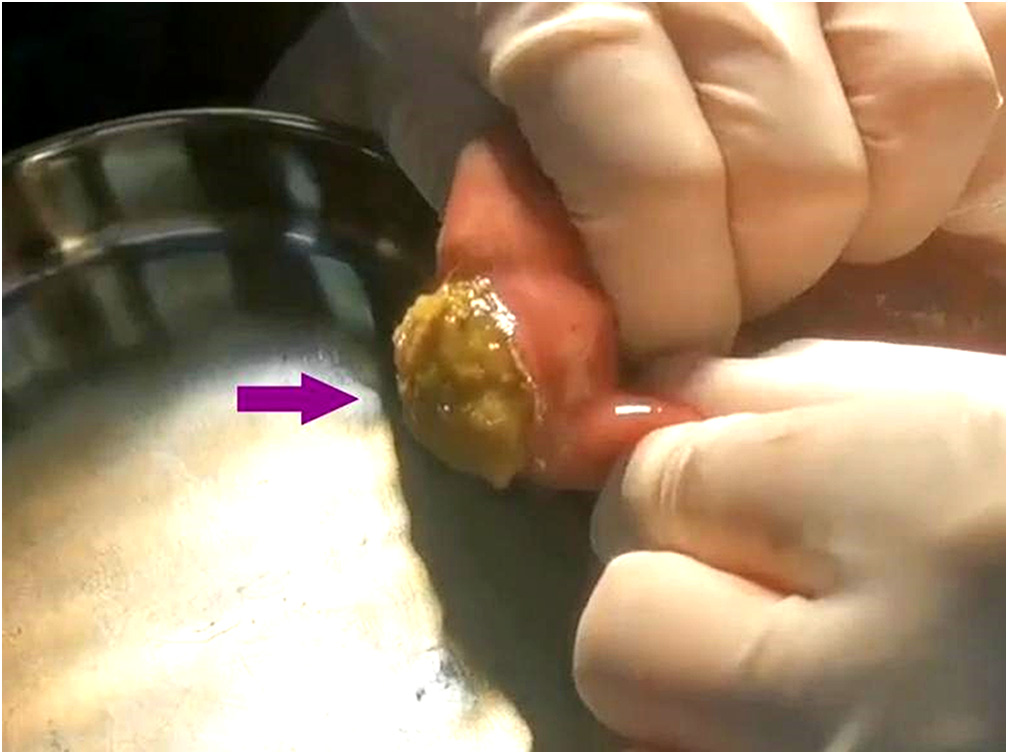
A longitudinal enterotomy was performed at the site of the bezoar in the small intestine, and the bezoar was then smoothly expelled.

**FIGURE 4:**
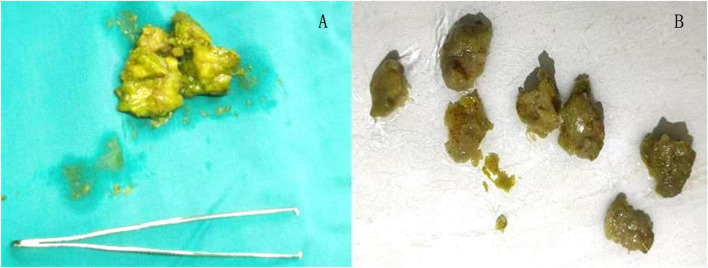
During the extraction process, the phytobezoar was fragmented **(A)**, and upon irrigation, it was identified as undigested pear **(B)**.

A 45-year-old female patient presented to the emergency department with a 5-day history of abdominal pain, nausea, and vomiting. The abdominal pain was colic-like, and abdominal distension was present; bowel movements and flatus were absent. The patient had a history of two cesarean sections and denied any previous GI issues. However, she reported the consumption of a large quantity of pears 2 days before the onset of symptoms. Physical examination revealed abdominal distension and tenderness upon palpation without rebound tenderness or muscular rigidity. Bowel sounds were diminished. Laboratory investigations revealed no significant abnormalities. A computed tomography (CT) scan showed several dilated small-bowel loops with fluid in the abdomen and a transition point in the right lower abdomen (Figure 1). The patient was diagnosed with SBO, and the possibility of a neoplasm was also considered. The patient was subsequently admitted for conservative management, which included fasting, intravenous fluid administration, antibiotics, and nasogastric tube decompression. Despite 28 h of conservative management, the patient’s symptoms persisted. Thus, we counseled the patient and the family in detail about the risks of continuing non-operative treatment—namely, bowel perforation, peritonitis, intestinal ischemia, surgical delay, symptom progression, and psychological stress while also explaining the potential risks and benefits of laparoscopic exploration. After careful consideration, the patient and family elected to proceed with laparoscopic surgery. No adhesions were observed during the surgical procedure, and a foreign body obstructing the intestinal canal was discovered approximately 60 cm from the ileocecal region. This obstruction resulted in significant dilation and fluid accumulation in the proximal intestinal canal, and the distal section collapsed (Figure 2). A 4-cm incision was made in the abdomen to access the obstruction site, and a longitudinal enterotomy was performed to extract the foreign body (Figure 3). The foreign body was identified as a bezoar measuring approximately 30 mm × 30 mm; the specimen fragmented into multiple pieces during extraction (Figure 4A). The bezoar was subsequently analyzed, revealing that it comprised undigested pear material (Figure 4B). The intestinal incisions were closed transversely, and the abdominal incisions were closed in a layered manner. The patient experienced an uneventful recovery and was discharged on the fifth postoperative day with dietary modification instructions to prevent future occurrences of gastrolithiasis. The patient showed no signs of recurrent SBO or surgery-related complications at the 3-month follow-up.

## Discussion

Small bowel obstruction caused by bezoars is rare, accounting for 0.4%–4.8% of all SBO cases. This observation is substantiated by more current literature, which states that SBO owing to bezoars occurs in roughly 2%–4% of patients (6, 7). The terminal ileum, located around 50–70 cm from the ileocecal valve, is the most prevalent site of obstruction due to its narrow lumen and limited peristalsis (8). Phytobezoars, which are made up of indigestible plant fibers, are the most common cause of such blockages over the world.

Persimmons are the most usually implicated source of phytobezoar production, notably the so-called diospyrobezoars due to their high tannin content, discovered even in patients without prior GI surgery (7). Other food sources implicated include celery, pumpkin, grape skins, prunes, oranges, coconut, sunflower seeds, watermelon seeds, and mushrooms. Pear bezoars, on the other hand, are extremely rare, with only a few occurrences documented in literature (9–12). Our report of a pear phytobezoar-induced small bowel obstruction (SBO) contributes to this limited body of information.

Predisposing factors for bezoar development include decreased stomach acidity, poor digesting, pyloric sphincter dysfunction, delayed gastric emptying, and previous gastric surgery (13). Systemic illnesses, including psychiatric problems, Crohn’s disease, hypothyroidism, diabetes, Guillain-Barré syndrome, GI tumors, and myotonic dystrophy, have been related to bezoar development. In our case, the patient had no history of stomach surgery or identified risk factors though she did eat quickly, which most certainly contributed. Furthermore, the high insoluble fiber (cellulose and pectin) in pear skin is consistent with the resistant nature of phytobezoars.

Patients with SBO due to bezoars often present with pain in the abdomen (96%–100%), nausea, vomiting, early satiety, upper abdominal discomfort, and occasionally weight loss (7). Our patient experienced growing stomach discomfort, distension, vomiting, and constipation, which mirrored the symptoms observed in Albostani et al.’s case of small-bowel obstruction caused by a meat bolus bezoar” (14).

The underlying etiology of SBO is difficult to detect clinically; hence, radiological imaging is required. Recent investigations confirm CT scans have a sensitivity of 73%–95% and specificity up to ∼60–100% for diagnosing phytobezoar-induced SBO by identifying intestinal edema, strangling, ischemia, and other obstructive characteristics (7). Ultrasonography has also demonstrated diagnosis rates of 88%–99% for bezoar-induced SBO, despite limitations caused by gas interference and operator dependency (8). In our case, contrast-enhanced CT performed the day before surgery showed excellent diagnostic clarity, supporting Albostani et al.’s claim that CT is the diagnostic gold standard for bezoar-induced SBO (14). This is further confirmed by Oh et al.’s results that the use of abdomen CT promotes correct preoperative diagnosis and allows for earlier surgical intervention (15).

The management of bezoar-induced SBO starts with vigorous fluid resuscitation and nasogastric decompression. Chemical dissolving agents such as saline, sodium bicarbonate, and, most notably, Coca-Cola have been successfully utilized to treat gastric bezoars. A systematic evaluation indicated that Coca-Cola alone completely dissolved phytobezoars in approximately 50% of cases, and when paired with endoscopic fragmentation, success increased to more than 90%. However, diospyrobezoars were less amenable only ∼23% dissolved with Coca-Cola alone—but further endoscopic procedures were successful in ∼84.6% of those cases. When pharmacological or endoscopic treatments fail, surgical intervention is required (16).

Brito et al. reported successful endoscopic clearance of small-bowel bezoars utilizing combined upper and lower GI endoscopy, indicating that non-surgical therapy can be helpful in certain instances (17). Nonetheless, surgical procedures are the only definitive treatment for bezoar-induced SBO, especially when conservative therapy fails or complications like ischemia or perforation occur. Recent research increasingly supports laparoscopic techniques as the preferred method for intestinal bezoar removal due to their minimally invasive nature, faster GI recovery, lower wound infection rates, and decreased anastomotic leaking when compared to open surgery. Surgeons should carefully evaluate the whole GI system for multiple bezoars to avoid recurring blockage (13, 18). While milking the bezoar distal to the ileocecal valve is an option, it increases the risk of serosal rips, mesenteric damage, and distal obstruction.

Conservative therapy failed in our patient; thus diagnostic laparoscopy was performed. To prevent intraperitoneal contamination, a 4-cm auxiliary incision was created to expose the afflicted small intestinal segment. An enterotomy was then performed, and the bezoar was removed intact. Because the bezoar was found distant to the ileocecal valve, manual advancement posed hazards of serosal rips, mesenteric damage, or distal obstruction; therefore, enterotomy was chosen as the safer way of extraction.

## Conclusion

This case highlights the importance of taking a comprehensive medical history, particularly regarding dietary habits and the consumption of phytobezoar-inducing foods, when diagnosing patients with intestinal obstruction and no history of prior surgery. Such an approach can help reduce the likelihood of misdiagnosis and ensure that patients receive timely and effective treatment. Additionally, while bezoars may be an uncommon and less familiar condition to many clinicians, maintaining a high index of suspicion in susceptible patients with GI symptoms is crucial for accurate diagnosis.

## Patient perspective

As a 45-year-old woman with no prior GI issues, I did not expect that eating pears would cause such a severe problem. I had consumed a lot of pears 2 days before my symptoms started. I experienced severe abdominal pain, nausea, vomiting, and abdominal distension for 5 days, and I could not pass gas or have bowel movements. At the emergency department, doctors found that my abdomen was distended and tender, with diminished bowel sounds. A CT scan showed a blockage in my small intestine. After 28 h of conservative treatment with no improvement, I had laparoscopic surgery. The doctors removed a bezoar made of undigested pear material from my terminal ileum. I recovered well and was discharged on the fifth day after surgery. The doctors gave me dietary advice to prevent recurrence. At my 3-month follow-up, I had no complications or recurrence. This experience taught me to be more cautious about my diet and to seek medical help promptly if I have similar symptoms in the future.

## Data Availability

The original contributions presented in this study are included in this article/supplementary material, further inquiries can be directed to the corresponding author.
